# Thermal ecology and baseline energetic requirements of a large‐bodied ectotherm suggest resilience to climate change

**DOI:** 10.1002/ece3.7649

**Published:** 2021-05-07

**Authors:** Hayley L. Crowell, Katherine C. King, James M. Whelan, Mallory V. Harmel, Gennesee Garcia, Sebastian G. Gonzales, Paul H. Maier, Heather Neldner, Thomas Nhu, John T. Nolan, Emily N. Taylor

**Affiliations:** ^1^ Biological Sciences Department California Polytechnic State University San Luis Obispo CA USA; ^2^ Ecology and Evolutionary Biology Department University of Michigan Ann Arbor MI USA; ^3^ Crocodile Lake Wildlife Refuge U.S. Fish and Wildlife Service Key Largo FL USA; ^4^ Jekyll Island Authority Conservation Department Jekyll Island GA USA

**Keywords:** climate change, ectotherm, energetic requirements, metabolism, rattlesnake, thermal ecology, thermal quality

## Abstract

1. Most studies on how rising temperatures will impact terrestrial ectotherms have focused on single populations or multiple sympatric species. Addressing the thermal and energetic implications of climatic variation on multiple allopatric populations of a species will help us better understand how a species may be impacted by altered climates.
2. We used eight years of thermal and behavioral data collected from four populations of Pacific rattlesnakes (*Crotalus oreganus*) living in climatically distinct habitat types (inland and coastal) to determine the field‐active and laboratory‐preferred body temperatures, thermoregulatory metrics, and maintenance energetic requirements of snakes from each population.
3. Physical models showed that thermal quality was best at coastal sites, but inland snakes thermoregulated more accurately despite being in more thermally constrained environments. Projected increases of 1 and 2°C in ambient temperature result in an increase in overall thermal quality at both coastal and inland sites.
4. Population differences in modeled standard metabolic rate estimates were driven by body size and not field‐active body temperature, with inland snakes requiring 1.6× more food annually than coastal snakes.
5. All snakes thermoregulated with high accuracy, suggesting that small increases in ambient temperature are unlikely to impact the maintenance energetic requirements of individual snakes and that some species of large‐bodied reptiles may be robust to modest thermal perturbations under conservative climate change predictions.

​

## Graphical abstract

We used long‐term thermal data collected from four populations of Pacific rattlesnakes (*Crotalus oreganus*) to determine and compare thermoregulatory metrics and maintenance energetic requirements of snakes living in climatically distinct habitats (coastal and inland). Thermal quality was best at coastal sites, but inland snakes thermoregulated more accurately despite being in more thermally constrained environments. Population differences in standard metabolic rate estimates were driven by body size rather than body temperature, with inland snakes requiring 1.6× more food annually than coastal snakes.

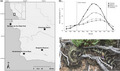

## 1 INTRODUCTION

The urgency of the biodiversity crisis is accelerating as scientists document how climate change, habitat loss, pollution, and other human‐induced disturbances are causing extinction of many taxa, such as insects, amphibians (Deutsch et al., 2008), mammals (Davies et al., 2008; Thuiller et al., 2006), birds (White & Bennett, 2015; Wormworth & Mallon, 2006), and reptiles (Böhm et al., 2013; Gibbons et al., 2000; Sinervo et al., 2010; Urban, 2015). As ectotherms, reptiles depend heavily on their immediate surroundings to regulate body temperature, where even slight changes in environmental conditions can impact physiological functions (Besson & Cree, 2010; Huey, 1982; Walther et al., 2002). Researchers have begun to quantify the current and predicted effects of climate change on diverse reptile species using recently available high‐resolution climate change forecasts and technology for modeling thermal landscapes (Böhm et al., 2016; Brusch et al., 2016; Sinervo et al., 2010; Wright et al., 2016).

Small‐bodied, heliothermic (sun‐basking) lizards are the focus of many climate‐based studies, largely because it is easy to obtain large sample sizes with limited effort and because these heat‐loving species may be at high risk of further warming (Buckley et al., 2015; Clusella‐Trullas et al., 2011; Pelegrin & Bucher, 2012; Sinervo et al., 2010). However, studies investigating thermal ecology in larger‐bodied ectotherms have tended to only use single populations of a given species and/or focus on sympatric species (Beck, 1995; Blouin‐Demers & Weatherhead, 2001; Blouin‐Demers & Weatherhead, 2002; Bovo et al., 2012; Lelièvre et al., 2011; Moore, 1978), potentially because these species tend to be less common, rendering the effort and expense involved in these studies prohibitive. This, in turn, limits the scope of these studies to certain localities and prevents inferences about the possibility that climate change and environmental variation will interact in their future impacts on a given species. Furthermore, many of these studies have focused on the direct impacts of altered temperatures on the body temperature (*T*_b_) of the population while failing to address the implications of changing *T*_b_ on the population's energetic needs (Alford & Lutterschmidt, 2012; Waldshmidt et al., 1986). To the best of our knowledge, no study to date has compared the thermal ecology and energetic requirements of a large‐bodied reptile across multiple populations that inhabit distinct thermal environments.

Here, we examine the thermal ecology of a large‐bodied reptile, the Pacific rattlesnake (*Crotalus oreganus*), across multiple populations while also extending our inference to quantify the energetic implications of environmental variation. The extraordinarily low metabolic rates and energetic allocation to specific physiological functions are well established in rattlesnakes (Beaupre & Duvall, 1998a, 1998b). We conducted intensive field studies collecting physiological and temperature data from four field sites on the Central Coast of California over eight years to quantify the thermal ecology (see Table S1 for explanations of terminology common in thermal ecology studies) and energy requirements of snakes on a macroecological scale. We then subjected these data to predicted increases in ambient temperature to examine how snake annual maintenance energy requirements will be impacted in a warming world. We hypothesized that precise thermoregulation and low metabolic rates allow rattlesnakes to respond to variable thermal environments effectively, both now and in the future due to climate change. At low temperatures, the snakes expend very little energy, but as temperatures rise, their precise thermoregulation allows them to remain at body temperatures optimally suited for their physiological processes. Specifically, we predicted that the thermal quality of habitats would differ, with hot and thermally variable inland sites having poorer thermal quality than the cool and stable coastal sites. Additionally, due to the climatic differences between these habitat types, we predicted that coastal snakes would have lower field‐active *T*_b_ and therefore lower annual maintenance energy expenditures than snakes at inland habitat, making them less thermally constrained both currently and in the future.

## 2 MATERIALS AND METHODS

### 2.1 Study species

The Pacific Rattlesnake (*Crotalus oreganus*, Holbrook 1840) ranges in western North America from southern British Columbia to Baja California, Mexico (Pook et al., 2000; Sunagar et al., 2014). The taxonomy of this species is under debate, and our four study sites fall into what is currently considered the integration zone of the northern (*C. o. oreganus*) and southern (*C. o. helleri*) subspecies (Ashton & Queiroz, 2001). However, recent evidence suggests that all these study populations genetically cluster (Holding et al., 2021); for the purpose of this study, we will refer to them as *C. oreganus*. They are habitat and dietary generalists that prey primarily on small mammals and lizards (Mackessy et al., 2003; Sparks et al., 2015; Sunagar et al., 2014). Body size varies widely among localities, but typical snout–vent lengths (SVLs) of adult male *C. oreganus* in California range from approximately 60 cm to 120 cm (Aldridge, 2002; Ashton, 2001).

### 2.2 Study sites

**FIGURE 1:**
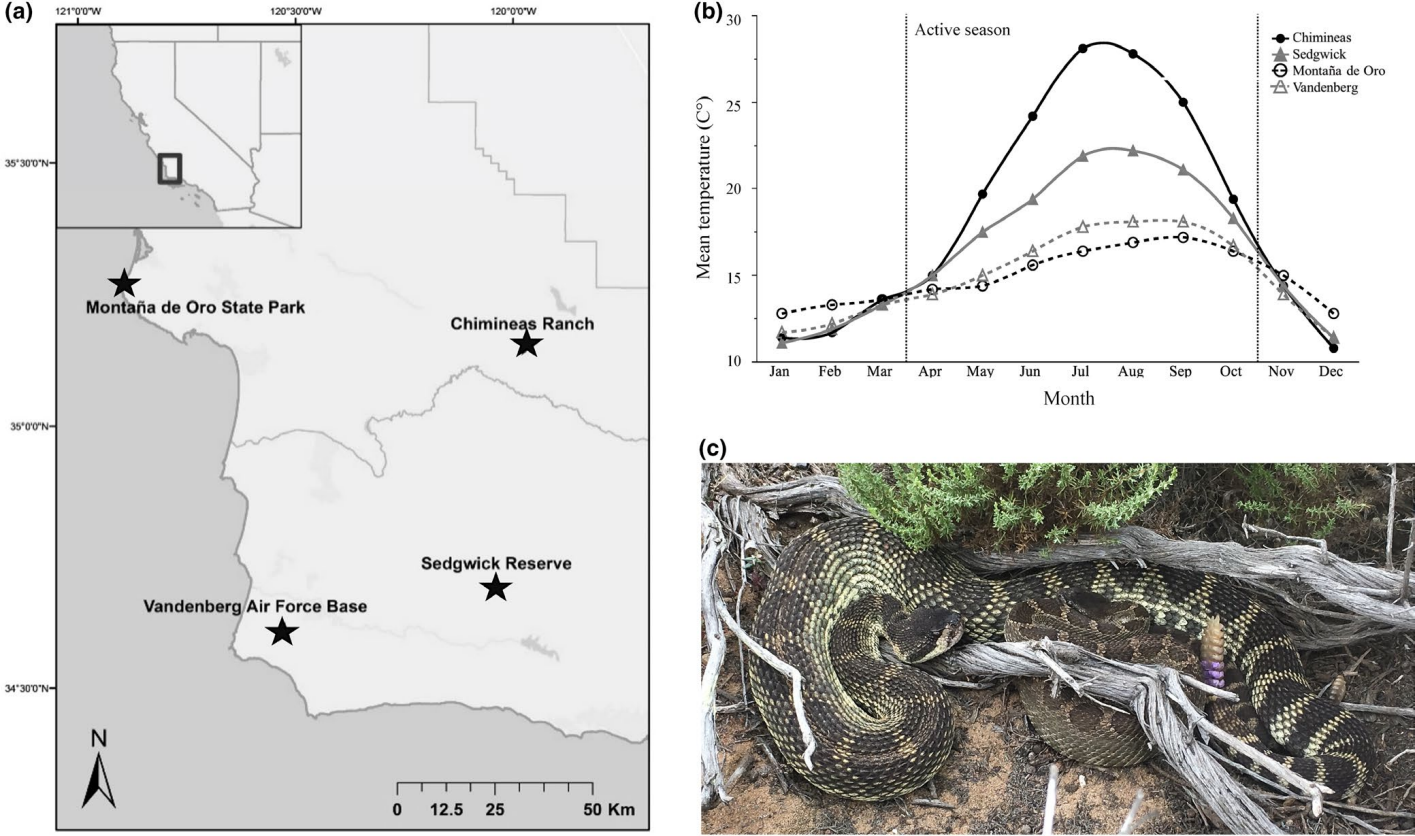
(a) Map of locations on the Central Coast of California of the four Pacific rattlesnake (*Crotalus oreganus*) populations (two coastal, two inland) used in this study. (b) Average monthly temperatures (°C) for the same corresponding sites from 2010 to 2017 (National Ocean and Atmospheric Administration & Western Regional Climate Center, 2017). (c) An adult male *Crotalus oreganus* (larger snake) resting with a smaller female mate

The four study sites used for this investigation were the Chimineas Ranch in the Carrizo Plain Ecological Reserve (CR), Montaña de Oro State Park (MDO), the University of California Sedgwick Reserve (SG), and Vandenberg Air Force Base (VAFB; Figure 1a). MDO and VAFB are coastal sites characterized by rugged cliffs, canyons, and coastal scrub plant communities that experience relatively stable and mild seasonal temperatures (Figure 1b; Capehart et al., 2016; Underwood et al., 2003). CR and SG are inland sites that experience higher and more variable daily and seasonal temperatures (Figure 1b) and are dominated primarily by chaparral, oak savanna, and grassland plain habitats (Chimineas Ranch Foundation, 2019; University of California Reserve System: Natural Resources, 2019). CR and MDO are situated to the north and are in San Luis Obispo County, CA, USA, whereas SG and VAFB are to the south in Santa Barbara County, CA, USA, (Table S2).

### 2.3 Preferred body temperature (*T*_set_)

We constructed a laboratory thermal gradient adapted from Bovo et al. (2012), spanning a range of ecologically relevant temperatures from 8–46°C to determine preferred body temperature (*T*_set_) (see Supplemental Methods; *Thermal Gradient Construction & Data Collection*). Snakes (*N* = 45) of varying size (35 cm–108.4 cm) and sex (*M* = 41, *F* = 4) were collected from all four field sites opportunistically from September 2017 to June 2018 (Table 1). Snakes were brought back to the California Polytechnic State University (Cal Poly) campus, where basic morphometrics (mass(g) and SVL(mm)), sex, reproductive status, and presence of gut contents were recorded. Snakes were excluded from the analysis if they were found to have visible meals in their gut or detectable follicles/offspring, as these factors could dramatically alter preferred temperatures. Snakes were placed in the thermal gradient for a two‐hour acclimation period (Bovo et al., 2012) followed by a 12‐hr data recording period. We defined *T*_set_ as the interquartile range of the data (Blouin‐Demers & Weatherhead, 2001; Fitzgerald et al., 2003). Data collected for this and the following components of this project were analyzed in JMP v14.0 (JMP^®^, SAS Institute Inc., Cary, NC, USA. 1989–2007) unless otherwise specified. Site, sex, mass, season (Charland et al., 1990), and presence/absence of internal radio‐transmitter (see below) were evaluated as predictor variables in the model examining the response variable median *T*_set_ (Blouin‐Demers & Weatherhead, 2001; Fitzgerald et al., 2003).

**TABLE 1:** Summary mass and sex data of Pacific rattlesnake (*Crotalus oreganus*; *N* = 45 snakes) used for preferred body temperature (*T*_set_) trials, range of temperatures reached during trials, and median site *T*_set_

Site	*n*	No. of individuals with radio‐transmitters	Sex	Mass range (g)	*T*_set_ Range (℃)	Mean median *T*_set_
CR (inland)	8	3	*M* = 5 *F* = 3	370–790	30.0–36.3	32.25
SG (inland)	11	5	*M* = 10 *F* = 1	347–912	11.7–36.6	26.47
MDO (coastal)	15	6	*M* = 15	60–860	14.8–37.6	30.55
VAFB (coastal)	11	3	*M* = 11	165–990	15.8–36.7	27.74

### 2.4 Field‐active body temperatures (Tb)

Between the years of 2010–2017, we collected 85 adult, male rattlesnakes across each of the four study sites (CR = 23, SG = 28, MDO = 15, VAFB = 19; Table S3). We studied snakes at one site in each of 2010–2016 (CR: 2010, SG: 2015, MDO: 2014, VAFB: 2012, 2013), and we studied snakes simultaneously at all four sites in 2017. Subjects were transported to the laboratory at California Polytechnic State University (San Luis Obispo, California, USA) and surgically implanted with radio‐transmitters (Holohil models SB‐2, 5.2 g and SI‐2, 11 g, 13.5 g; Holohil Systems Ltd., Carp, Ontario, CA) and Thermochron iButtons (DS1922L‐F5 and DS1921G‐F5 models, accuracies = ±0.5°C and ±1°C respectively, Maxim Integrated Products Inc., Rio Robles, San Jose, CA), which were set to record field‐active body temperatures (*T*_b_) every hour. Temperature loggers and radio‐transmitters were implanted intracoelemically as separate units following the procedures of Claunch et al. (2017). Snakes were released within 1–2 days of surgery at the location of capture. At the end of each active season, we recaptured snakes and brought them back to the laboratory to remove iButtons and radio‐transmitters. To compare the mean field‐active *T*_b_ of snakes across the four sites, we used a mixed‐effects model with an AR(1) covariate structure to capture the serial correlation of the within‐snake observations of temperature (Millar & Anderson, 2004). Site, month, and time of day acted as fixed effects, snake ID was a random effect, and a unique time of day/date value for each data point was used for the repeated structure. Additional sin‐ and cos‐transformed time‐of‐day variables were also included as fixed effects to account for the cyclic, continuous nature of time.

### 2.5 Snake physical model temperatures (*T*_mod_)

In 2017, we deployed physical models in exposed, shaded, and burrow microhabitats throughout the four field sites to characterize the thermal landscapes potentially available to each of the rattlesnake populations (Dzialowski, 2005; Lutterschmidt & Reinert, 2012). Physical model microhabitat sites were selected based on previous observations of snake use within those areas and also with an effort to distribute them evenly throughout the area where snakes were being radiotracked. Importantly, these models represent a range of low and high temperatures available to the snakes, but do not encompass the relative amount of each microhabitat available to snakes at each field site. Physical models consisted of water‐filled copper pipes, painted to approximate *C. oreganus* reflectance, with iButtons suspended inside in accordance with Bakken (1992) and Lutterschmidt and Reinert (2012) (see Supplemental Methods; *Physical Model Construction & Validation*). Model temperature (*T*_mod_) was recorded every hour for one year from June 2017 to June 2018. Five physical models were placed at each of the four study sites in various microhabitats (Blouin‐Demers & Weatherhead, 2002; Lutterschmidt & Reinert, 2012): two inside typical snake refugia (e.g., ground squirrel burrows, rock burrows), two in exposed habitat (open field, gravel road), and one in a shaded habitat (under a shrub or bush). We validated models using similar‐sized, adult rattlesnake carcasses. Because our models contained water (therefore had thermal inertia), they did not meet the definition of an operative temperature model (Bakken & Gates, 1975). Rather, we built our models to have thermal properties similar to those of a nonthermoregulating snake, meaning that the temperatures collected by our models reflected the full range of maximum high and low temperatures and average, adult‐size animal could reach if it were to remain in the most thermally stable and most thermally variable microhabitats within each of the four study sites. For a large‐bodied, nonheliothermic, nonactive shuttling ectotherm, we believe that these models were most accurate for quantifying the thermal conditions of each habitat as opposed to smaller, air‐filled models (Lutterschmidt & Reinert, 2012).

To compare *T*_mod_ values across different sites, we used a mixed model with an AR(1) covariate structure similar to the model used for *T*_b_ (see above section on body temperature). No *T*_mod_ values for exposed microhabitats for SG were included because the *T*_mod_ exceeded iButton temperature limits, causing all exposed models at SG to fail. Additionally, *T*_mod_ values for the shaded CR model are unavailable for months July–December due to iButton failure. Site, month, and time of day were included as fixed effects.

### 2.6 Thermal ecology variables measured

We used thermal indices developed by Hertz et al. (1993) to evaluate the extent to which a given habitat temperature (*T*_mod_) permits a *T*_b_ within *T*_set_ to be achieved (thermal quality) and the extent to which an animal actually experiences *T*_b_ within its *T*_set_ (thermoregulatory accuracy). We calculated thermal quality of the environment (*d*_e_) as the absolute value of the difference between *T*_mod_ and *T*_set_, where high *d*_e_ values mean that the thermal quality of the environment is low and *d*_e_ values approaching zero represent more favorable thermal habitat. The metric of *d*_e_ by definition uses operative temperature (*T*_e,_ a value obtained from physical models without thermal inertia instead of the metric *T*_mod_); however, we use *T*_mod_ here with the caveat that the models' inertia could impact their cooling and heating rates. We calculated thermoregulatory accuracy (*d*_b_) as the absolute value of the difference between *T*_b_ and *T*_set_, where high *d*_b_ values mean poor thermoregulatory accuracy (i.e., the snake's actual body temperature is much higher or lower than its *T*_set_), and *d*_b_ values approaching zero represent accurate thermoregulation (Blouin‐Demers & Weatherhead, 2001; Hertz et al., 1993). Calculation of thermal variables was performed in R v. 3.4.4 (R Development Core Team, 2015) using the package “dplyr” (François et al., 2018).

Individual *T*_mod_ values for physical models of the same site and type (for example, all CR burrow models) were averaged to calculate a mean *d*_e_ value for each time of day observation (hr) for each month. A repeated‐measures ANOVA was conducted to compare overall *d*_e_ values of each study site and then rerun with results blocked by physical model (burrow, shaded, exposed) to examine differences in thermal quality among microhabitat types. Because of the need to compare multiple levels/groups (both site and microhabitats within site), the repeated‐measures analyses with the AR(1) covariate structure was not used for *d*_e_. Site, month, and time of day were included as fixed effects in this model as well as their interactions in a full factorial to account for these variables. For *d*_b,_ because we were only examining differences at the site level, we used the same statistical analysis that we used for *T*_b_ and *T*_mod_ values (see above sections) with site, month, and time of day included as fixed effects.

### 2.7 Energetics

We used snake field‐active body temperatures (*T*_b_) and morphometric data to calculate theoretical standard metabolic rates (SMR) based on the following equation established by Beaupre and Duvall (1998b, see Supplemental Methods: *Energetics*):

$$\text{SMR =}\;\log_{10}{\text{VO}}_{2}=X_{1}+\log_{10}\text{mass}+X_{2}*\text{temperature}+X_{3}$$

We converted the inverse log of the SMR into annual maintenance energy requirement in Joules (19.874 J/ml O_2_) and then Calories (2.3900 × 10^–4^ kcal/J). We then calculated the approximate annual prey requirements to meet maintenance costs of an average‐sized male rattlesnake for each of the four study sites using their most common prey item, the California ground squirrel (*Otospermophilus beecheyi*; Rowe & Owings, 1990; Sparks et al., 2015). Based on previously published food assimilation experiments in the genus *Crotalus* (Beaupre & Zaidan, 2012; Secor & Nagy, 1994), we assumed an 80% energetic assimilation efficiency and that an average, adult ground squirrel weighs approximately 500 g (Evans & Holdenried, 1943) and contains roughly 690 kcal (Dorcas et al., 2004; Kaufman et al., 1975). An ANCOVA was performed to compare the daily SMR (ml O_2_ day^−1^) of the four populations of snakes (*N* = 85) during their active season (April–October). Snake mass, site, and the site x mass interaction were included in the model. Because mass was used to estimate SMR, it will inevitably be a significant predictor variable for SMR. However, we included it in the models to account for variation in snake body size among sites.

### 2.8 Climate change projections

We used the California Energy Commission (2019) representative concentration pathway (RCP) climate scenario 4.5 as a “best case” scenario (emissions peak around the year 2040 then steadily decline) to estimate the changes in habitat thermal quality and therefore energetic consequences of anthropogenic climate change on snakes. We used the “modeled projected annual mean” tool to identify the years in which the annual average temperatures increase one degree from the 2017 average for each of the four study sites (CR/SG/VAFB = 2030, MDO = 2047). We repeated this procedure for a two‐degree increase as well. To make macroecological predictions, we then assumed that a 1°C increase in annual average temperature would be equivalent to the same increase in *T*_mod_ of all microhabitats. We calculated the proportion of current mean hourly *T*_mod_ for each site and microhabitat type that fell within *T*_set_ for 2017 as well as with 1°C and 2°C increases in mean hourly temperatures. We then calculated the percent change in these proportions between each of these three climate scenarios as well as the change in mean *d*_e_ for each site and microhabitat type. Lastly, we calculated the mean increase in annual energetic needs (kcal/year) assuming snake *T*_b_ increased along with *T*_mod_ by adding 1°C and 2°C to the mean hourly *T*_b_ of each snake and using the Beaupre and Duvall (1998b) equation to recalculate mean SMR for each of the four sites. We used a repeated‐measures ANOVA to compare differences in current energetic needs and those projected with 1°C and 2°C increases, with site included as a factor.

## 3 RESULTS

### 3.1 Preferred body temperature (*T*_set_)

The mean of the median *T*_set_ of all snakes (*N* = 45) was 29.22 ± 0.92°C with a 50% interquartile range of 26.28 ± 1.01°C ‐ 32.34 ± 0.84°C (Blouin‐Demers & Weatherhead, 2001). None of the factors tested (site, sex, mass, season, or presence of internal radio‐transmitter) significantly affected median *T*_set_ (*F*_8.36_ = 1.30, *p* = .27; Figure S1).

### 3.2 Field‐active body temperature (*T*_b_)

**FIGURE 2:**
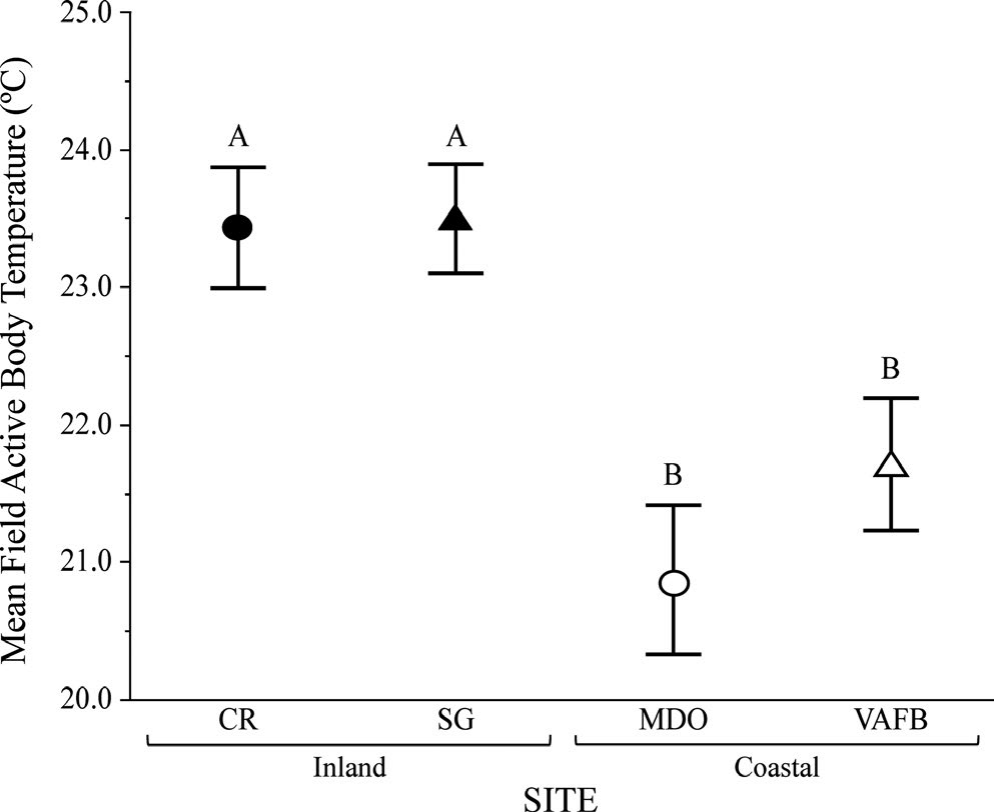
Mean hourly field‐active body temperatures (*T*_b_) for four populations of Pacific rattlesnake (*Crotalus oreganus*; *N* = 85; CR = 23, SG = 28, MDO = 15, VAFB = 19) on the Central Coast of California during the active season (April–October). Black shapes denote inland sites, white shapes denote coastal sites, circles denote northern sites, and triangles denote southern sites. Data were collected for varying lengths of time from 2010 to 2017. Different letters represent a significant difference between means, and bars represent 95% confidence intervals

After accounting for monthly and diel variation in temperature, we found that *T*_b_ differed significantly among sites, with snakes from both CR and SG having higher mean body temperatures than snakes from both MDO and VAFB (*F*_3,83.2_ = 26.16, *p* < .0001). Tukey–Kramer post hoc tests showed no significant differences in *T*_b_ between the two coastal populations or between the two inland populations (Figure 2).

### 3.3 Physical model temperatures (*T*_mod_)

**FIGURE 3:**
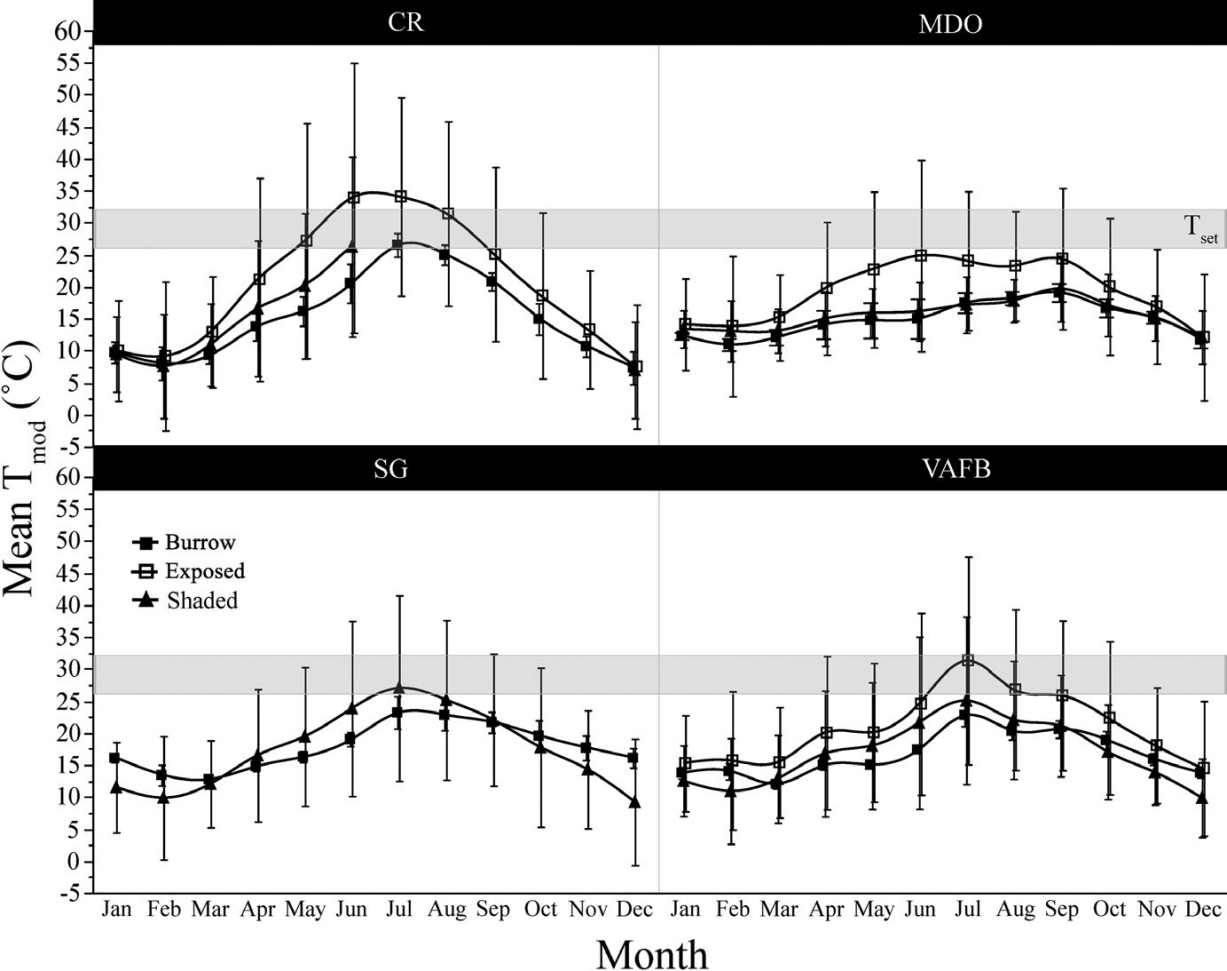
Mean monthly Pacific rattlesnake (*Crotalus oreganus*) physical model temperatures (*T*_mod_) for each of the four study sites (inland: CR, SG; coastal: MDO, VAFB) on the Central Coast of California (measured June 2017–June 2018). Mean monthly *T*_mod_ differed significantly among sites but overall annual *T*_mod_ did not. SG does not include exposed physical model values, as all exposed SG models failed, as well as the CR shaded model from months July–December. Gray bar represents the preferred body temperature range (*T*_set_) of *C. oreganus*. Error bars represent 1 ± standard deviation from the mean

After accounting for monthly and diel variation in temperature, we found that mean monthly *T*_mod_ differed significantly among sites (*F*_11_ = 366.18, *p* < .0001; Figure 3) although overall annual *T*_mod_ did not (*F*_3_ = 0.41, *p* = .75).

### 3.4 Thermal quality (*d*_e_)

**FIGURE 4:**
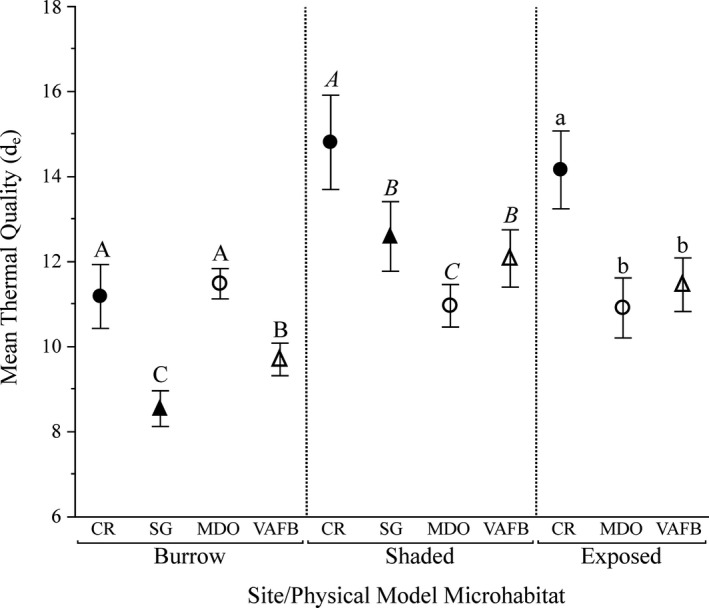
Mean thermal quality (*d*_e_) values for each microhabitat at each of four study sites (inland: CR, SG; coastal: MDO, VAFB) from June 2017 to June 2018. Thermal quality values closer to zero are indicative of better thermal quality. Different letters indicate significant differences among physical models in the same microhabitat type (capital letters = burrows, italicized = shaded, lowercase = exposed). SG does not include exposed physical model values, as all exposed SG models failed. Values are shown with 95% confidence intervals

A total of 3,048 mean hourly *d*_e_ values were obtained from physical models (*n* = 5/site) at all sites. There was a significant difference in *d*_e_ among study sites (*F*_1151,1896_ = 3.62, *p* < .0001); post hoc analysis showed no significant difference in overall mean *d*_e_ among the two coastal sites (MDO & VAFB) and SG but did show that mean CR *d*_e_ was significantly higher (= poorer thermal quality) than all other sites. Almost all interaction terms within the full factorial model were significant (Table S4). When the same test was run with *d*_e_ values blocked by microhabitat type, there was still a significant difference in *d*_e_ among sites within each microhabitat (burrow, *F*_3,1,114_ = 137.29, *p* < .0001; shaded, *F*_3,994_ = 75.13, *p* < .0001; exposed, *F*_2,827_ = 19.38, *p* < .0001; Figure 4). Because of the failure of SG exposed physical models, no exposed *d*_e_ values were included for the overall calculations for SG, therefore resulting in a lower overall *d*_e_ value than the expected actual value and reducing the SG model sample size to *n* = 3. Due to similar ambient temperatures, we expect that SG exposed *d*_e_ values would have been similar to CR exposed values.

### 3.5 Thermoregulatory accuracy (*d*_b_)

**FIGURE 5:**
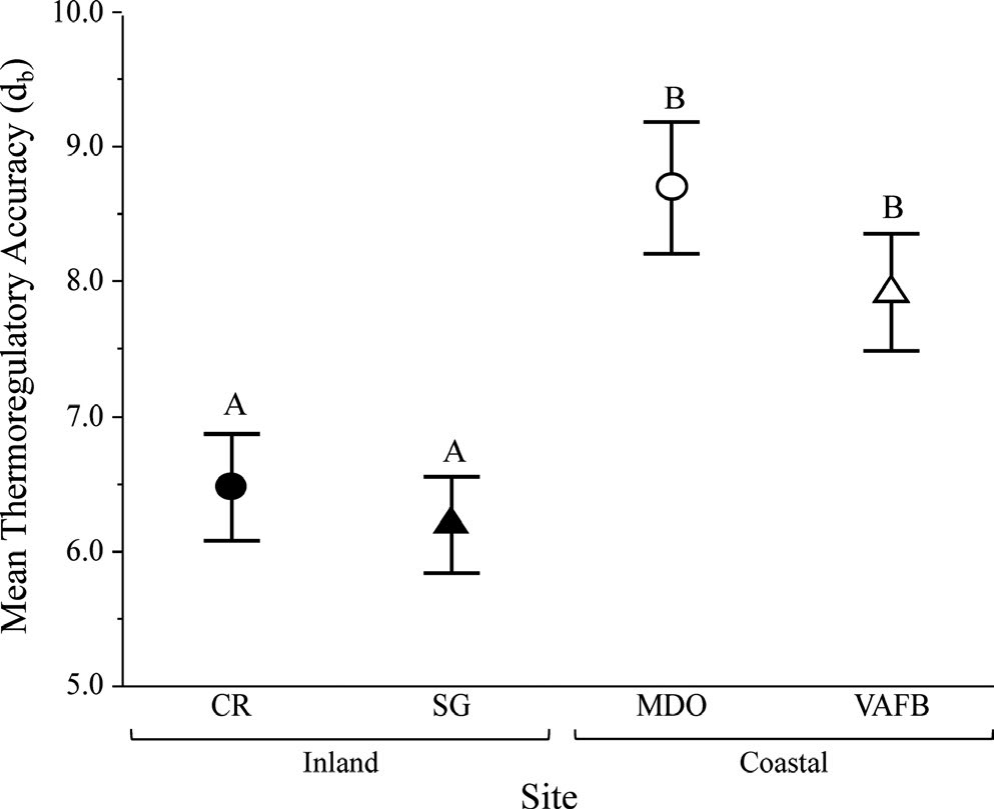
Mean thermoregulatory accuracy (*d*_b_) for each of four populations of Pacific rattlesnake (*Crotalus* oreganus; *N* = 85; CR = 23, SG = 28, MDO = 15, VAFB = 19) on the Central Coast of California. Black shapes denote inland sites, white shapes denote coastal sites, circles denote northern sites, and triangles denote southern sites. Values closer to zero reflect higher thermoregulatory accuracy. Mean daily *d*_b_ differed significantly among sites, with inland snakes (CR, SG) thermoregulating more accurately than coastal snakes (MDO, VAFB). Different letters represent significant differences between means, and bars represent 95% confidence intervals

**FIGURE 6:**
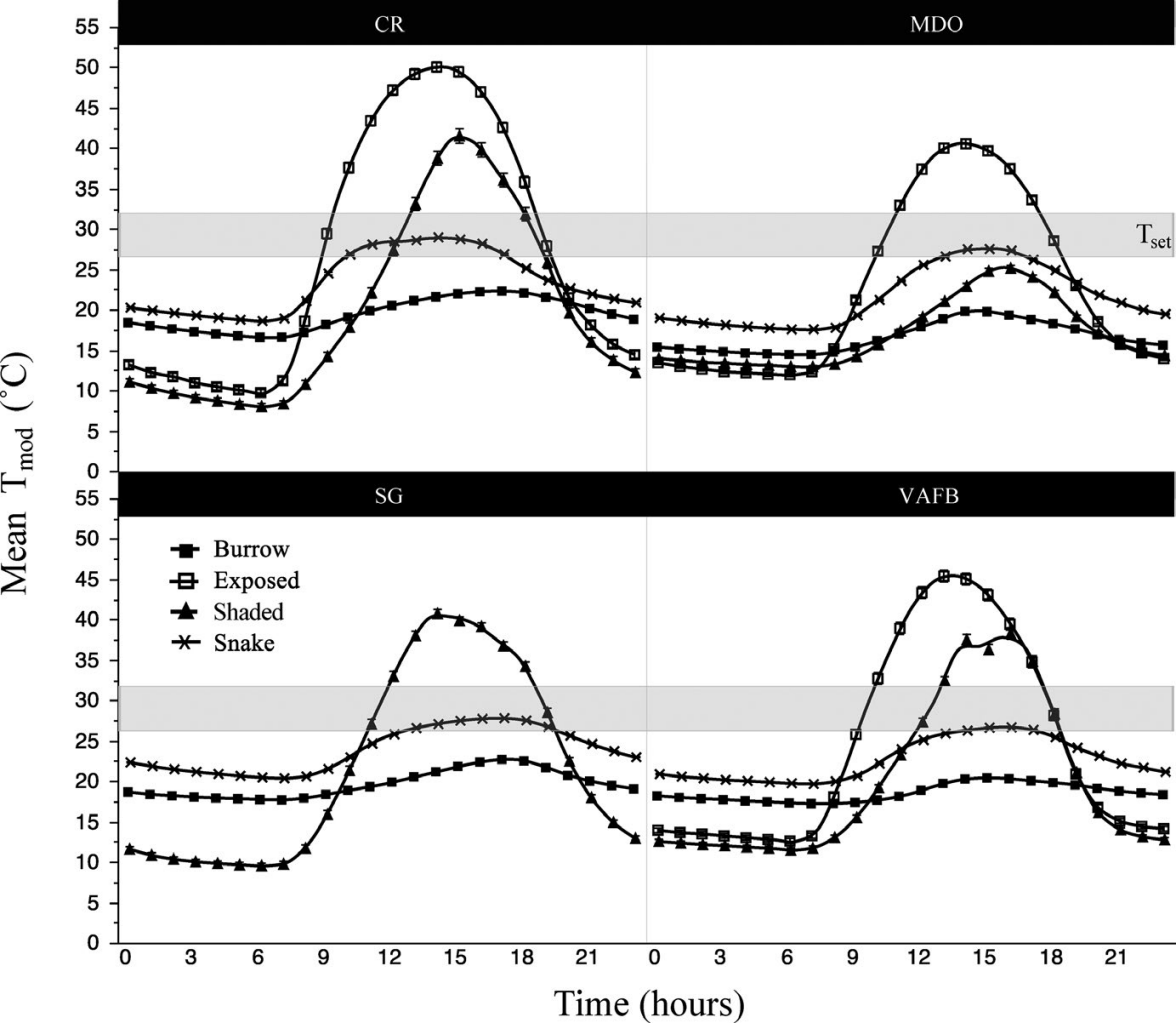
Mean hourly physical model temperatures (*T*_mod_, in burrows, exposed, or shaded) and Pacific rattlesnake (*Crotalus oreganus*) body temperatures (*T*_b_) over the duration of the active season (April–October) for each of the four study sites (inland: CR, SG; coastal: MDO, VAFB) on the Central Coast of California. CR shaded means do not include data from July–December. Gray bars represent the preferred body temperature range (*T*_set_) of *C. oreganus*

Thermoregulatory accuracy (*d*_b_) differed among study sites, with inland snakes (CR, SG) being more accurate thermoregulators than coastal snakes (MDO, VAFB; *F*_3_,_84.4_ = 37.17, *p* < .0001; Figure 5). Snakes at both CR and SG spent an overall larger portion of time in or near preferred body temperatures throughout the entirety of the study (Figure 5). On average, 50.89% of hourly *T*_b_ observations fell within the *T*_set_ for inland snakes versus only 21.63% for coastal snakes. Post hoc tests revealed no significant differences between the two inland sites or between the two coastal sites (Figure 6).

### 3.6 Energetics

**FIGURE 7:**
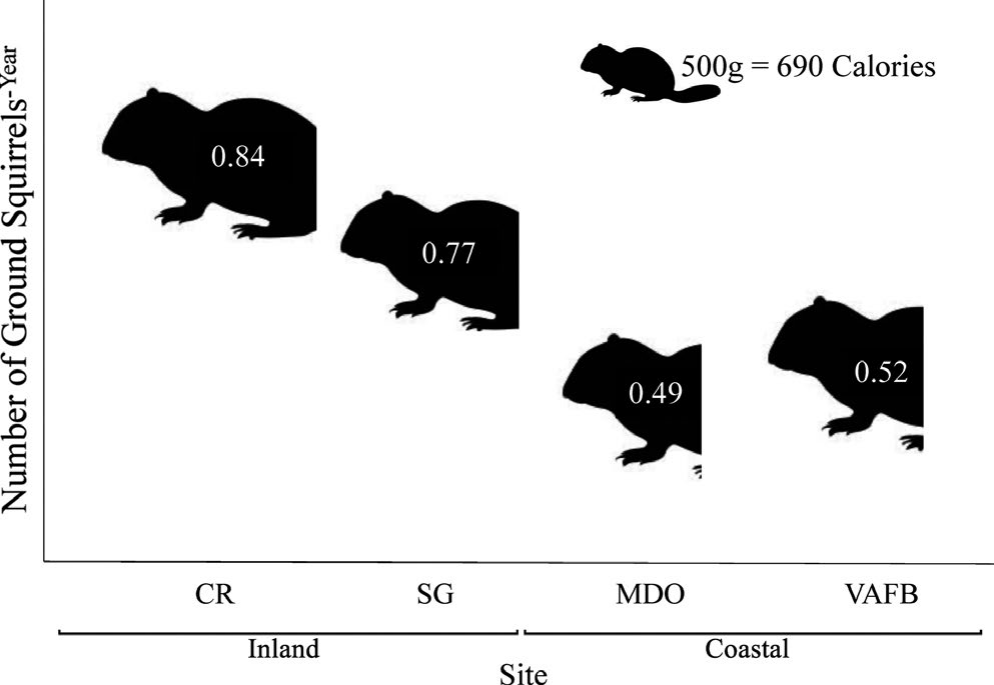
Number of California ground squirrels (*Otospermophilus beecheyi*) an average‐sized adult male Pacific rattlesnake (*Crotalus oreganus*) from each of four populations on the Central Coast of California (CR, SG, MDO, VAFB) would need to consume to meet annual maintenance energetic requirements

The daily SMR of snakes across all four study sites differed significantly, with inland snakes (CR, SG) having higher overall SMR than coastal (MDO, VAFB) snakes (*F*_7,77_ = 136.68, *p* < .0001, Figure S2). We suspect that the effect of site on SMR was not due to *T*_b_ differences among sites, but to body size differences (inland snakes are larger, Table S5). When correcting for mass (by dividing out mass from overall SMR), we found no significant site differences among SMRs (*F*_3,81_ = 1.50, *p* = .22). However, given that the goal of this study is to examine overall differences in SMR and energetic requirements, we will only focus on whole‐animal values as nonmass‐corrected data will be most informative for our initial questions (Lighton & Halsey, 2011). When mean SMR values were converted to annual energetic needs, we found that individual snakes from all four populations needed to eat less than the equivalent of one adult ground squirrel year^‐1^ to satisfy maintenance energetic requirements. An average‐sized, adult male inland snake would need to consume a mean of 0.80 ground squirrels per year whereas a coastal snake would need an average of 0.51 (Figure 7).

### 3.7 Climate change projections

**FIGURE 8:**
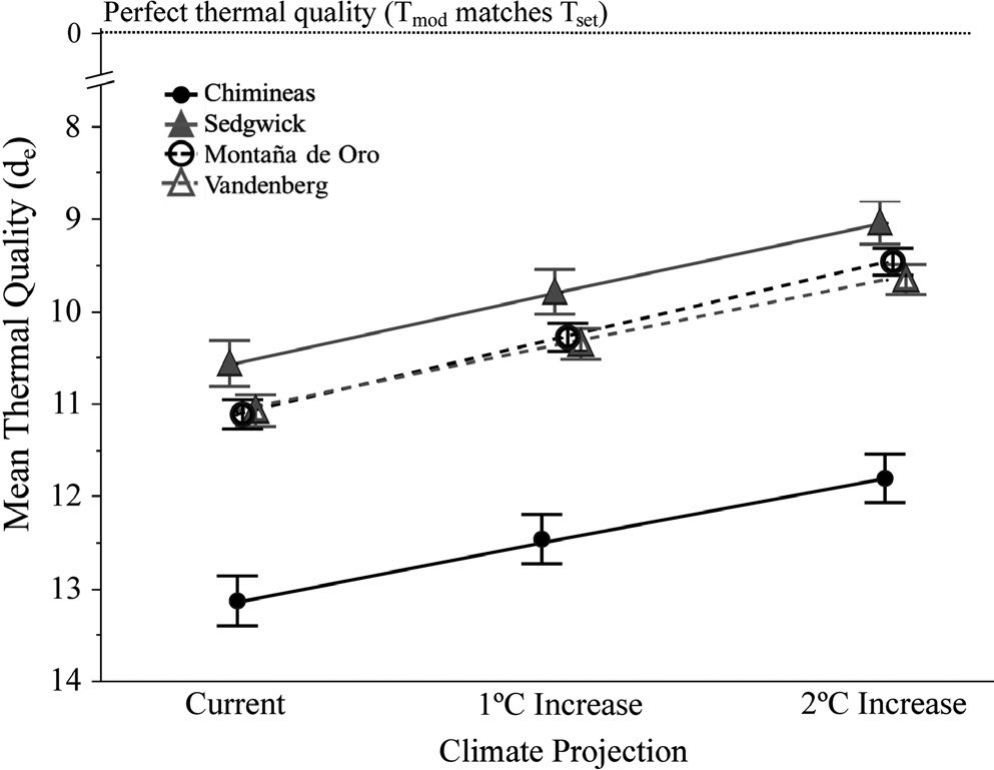
Overall thermal quality (*d*_e_) for four study sites (CR, SG, MDO, and VAFB) on the Central Coast of California at current conditions and with a 1°C and 2°C increase in ambient temperatures. Values approaching zero represent higher thermal quality. All sites will experience an increase in *d*_e_ as ambient temperatures increase, creating potentially more favorable thermal environments for Pacific rattlesnakes (*Crotalus oreganus*) in both coastal and inland locations. Error bars represent ± 1 *SEM*

During the physical model deployment period (June 2017–June 2018), the overall proportion of hourly *T*_mod_ that fell below *C. oreganus* *T*_set_ was considerably higher (0.856) than the proportion that fell within (0.056) or above (0.085) this range. While these proportions varied among and within sites as well as microhabitat types (Table S6), this general trend held true across all categories. With a 1°C increase in *T*_mod_, the overall mean proportion of hourly temperatures that fell within *T*_set_ increased to 0.064, with still the majority of hourly readings falling below *T*_set_ (0.084) and 0.091 falling above. A 2°C increase shows the same pattern, with a higher proportion of *T*_mod_ falling within the *T*_set_ range (0.075) than the previous two climate conditions, the proportion below *T*_set_ decreasing (0.825), and the proportion above *T*_set_ increasing (0.096, Figure S3). Additionally, thermal quality (*d*_e_) of each microhabitat type and the overall thermal quality of each site are projected to improve with increases in ambient temperature (Figure 8). With the greatest increase of 2°C, CR, SG, MDO, and VAFB will, respectively, experience an overall 10, 12, 13, and 11% increase in *d*_e_. These data suggest that rising temperatures associated with anthropogenic climate change could actually benefit *C. oreganus* as the thermal quality of their habitats increases.

Maintenance energy requirements (kcal/year) were overall significantly higher when incorporating annual increases of 1°C and 2°C in mean snake *T*_b_ (*F*_5,6_ = 106.97, *p* < .00001). However, post hoc tests revealed that whole model significance is driven by the differences between current and 1°C requirements, and requirements incorporating the 2°C increase. There are no significant increases from current energetic requirements to energetic requirements with an increase in snake *T*_b_ of 1°C (8.4% increase in annual maintenance energetic requirements). With a 2°C increase from current snake *T*_b_, mean energetic requirements increased by another 17.6%, which was a significant increase from the current and 1°C requirements. However, when converted to mean number of squirrels per year, all populations still required roughly 1 or less large meal (Table 2).

**TABLE 2:** Mean number of ground squirrels required by an average‐sized, adult male Pacific rattlesnake (*Crotalus oreganus*) from four populations on the Central Coast of California required to meet annual maintenance energy requirements at current body temperature (*T*_b_) conditions and with theoretical 1°C and 2°C increases in *T*_b_

Site	Current no. required squirrels	No. required squirrels with 1°C increase	No. required squirrels with 2°C increase
CR (Inland)	0.84 ± 0.04	0.90 ± 0.04	1.06 ± 0.04
SG (Inland)	0.77 ± 0.04	0.86 ± 0.05	1.01 ± 0.05
MDO (Coastal)	0.49 ± 0.04	0.53 ± 0.05	0.62 ± 0.04
VAFB (Coastal)	0.52 ± 0.02	0.57 ± 0.02	0.68 ± 0.02

## 4 DISCUSSION

In this study, we demonstrate that populations of *C. oreganus* experience dramatically different climatic conditions but utilize thermal variation in microhabitats to thermoregulate such that differences in mean active *T*_b_ are minor and that these differences may not have major impacts on the snakes' overall energetic needs. As a moderately precise thermoregulator (Blouin‐Demers & Weatherhead, 2001), *C. oreganus* will likely be able to mitigate the effects of inhabiting low‐quality thermal habitat by altering thermoregulatory behaviors, and furthermore, climate change is predicted to increase the thermal quality of *C. oreganus* habitat. Thus, this rattlesnake species and others may be fairly resilient to and even positively impacted by changing climates.

The *T*_set_ range (26.28 ± 1.01°C ‐ 32.34 ± 0.84°C) was similar in all four populations of *C. oreganus* and is notably wide in range, potentially because, as habitat generalists and ambush foragers, rattlesnakes are adapted to experiencing a wide range of environmental temperatures (Alford & Lutterschmidt, 2012). Other vipers also exhibit high variation in *T*_set_ within and among species (Table S7). This large range of temperature preferences exemplifies the tendency of larger‐bodied ambush‐predator ectotherms to exhibit more eurythermic behaviors that are reflective of the environments in which they are found (Bovo et al., 2012; Brattstrom, 1965; Moore, 1969). Snakes from coastal sites had significantly lower *T*_b_ than snakes from inland sites, which we expected based on different ambient temperatures. However, variation in *T*_b_ may also be related to environmental trade‐offs impacting the thermoregulatory behavior, such as site differences in predation risk, as well as body size and thermal inertia (Putman & Clark, 2017), which in the case of our study is inextricably linked to source population. That is, inland snakes could experience higher *T*_b_ in part because their larger body sizes impart slower cooling rates (Stevenson, 1985). Regardless of site, both inland and coastal snakes typically exhibited field‐active *T*_b_ below their *T*_set_. Snakes often select ambush sites in shaded areas along small mammal trails in grass or near ground squirrel burrows (Putman et al., 2016; Theodoratus & Chiszar, 2000) and remain for an extended period of time until a prey item passes within striking distance. Lower *T*_b_ resulting from ambush site selection may represent a thermoregulatory trade‐off as snakes passively thermoconform to low‐quality ambush sites (Alford & Lutterschmidt, 2012).

Our prediction that coastal sites would have overall better thermal quality than inland sites was supported (if we assume that SG would have a similar thermal quality to CR with the inclusion of values from exposed physical models), although there was no significant difference in annual overall mean *T*_mod_. The lack of overall difference in mean *T*_mod_ is likely due to the greater variance of temperatures in inland sites throughout the year relative to coastal sites, creating similar means. However, the variation in temperature among and within these sites is best demonstrated by the significant variation in thermal quality of each microhabitat. Overall, burrows have the best thermal quality due to their more stable temperatures, and it is likely that they offer cooler temperatures and further thermal stability deeper in the burrow systems which were unattainable to us for measurement. Snakes are able to use this microhabitat as dependable thermal refugia when surface temperatures are either too hot or cold. To our surprise, shaded physical models often experienced temperatures close to those of exposed models at their respective sites. It is possible that even though these models were shaded, the extreme heat of the inland sites and frequently windy and wet conditions of the coastal sites still drove the temperatures of these models far above and below *T*_set_ due to conduction and convection, respectively. Because thermal quality is more variable at inland sites, this may mean that snakes need to spend more energy shuttling between thermally favorable habitat during their active summer season as well as experience restricted daily and annual activity patterns. Meanwhile, the lack of large temperature fluctuations at coastal sites means snakes can remain active for a larger portion of daylight hours and for most, if not all of the year. Although physical models allowed us to produce a coarse estimate of the thermal landscape, we are unable to account for the entire thermal configuration of each site (Sinclair et al., 2016), and a more detailed analysis within each of these sites would help elucidate some of the drivers of differences in field‐active *T*_b_ observed in these populations of *C. oreganus*. Additionally, our study used environmental and body temperature data that only partially overlapped in study periods; future studies interested in making comparisons of fine‐scale relationships between microhabitat temperatures and snake body temperatures should collect data across the exact same time period, even if long‐term climates at study sites are relatively stable.

We were somewhat surprised to find that snakes in habitats with poorer thermal quality (inland sites) thermoregulated more accurately than snakes with access to higher quality habitats (coastal populations). While this phenomenon has been documented in multiple small lizard species (Gunderson & Leal, 2012; Sagonas et al., 2013), the number of studies reporting this in large‐bodied reptiles is limited (Besson & Cree, 2010; Blouin‐Demers & Weatherhead, 2001; Row & Blouin‐Demers, 2006), particularly in ambush predators (Bovo et al., 2012). It is possible that the higher thermal quality of coastal sites may result in longer periods of time where ambient temperatures are closer to the *T*_set_ of *C. oreganus*, putting less physiological pressure on snakes to actively thermoregulate. Additionally, snakes at the poorer quality inland sites may be thermoregulating more accurately to increase physiological performance for other behaviors (e.g., mate searching, ambush, etc.) during the more limited activity hours, as Besson and Cree (2010) reported in tuatara. The results may also reflect the thermal heterogeneity of the habitats: The thermal quality of microhabitats in inland sites was more variable than those in coastal sites (Figure 4), potentially affording inland snakes a wider range of choices when thermoregulating and allowing them to find ambush sites or refugia closer to their *T*_set_.

Our estimates of the SMR of snakes from all four populations revealed that snakes from inland sites require on average 1.6× as much food as coastal snakes for maintenance metabolism. The equations to estimate SMR (Beaupre & Duvall, 1998a, 1998b) use mass and *T*_b_; while both of these were higher at inland sites, the larger body size of inland males was the major contributor to their higher SMR and therefore energy requirements. Our data show that high variation in ambient temperatures among sites translates into only minor interpopulation differences in *T*_b_ due to effective thermoregulation and that these differences do not have a great impact on maintenance energy requirements. The driving factor for differences in overall metabolic rates, and therefore energetic needs, is the actual mass of the animal (Dorcas et al., 2004). Why are males larger at inland sites? There are many possibilities, including a warmer active season promoting a longer growing season (Mousseau, 1997), higher rainfall and water availability at our inland sites offsetting the negative effects of living in semi‐arid habitats (Amarello et al., 2010), more competition for resources at coastal sites due to higher rattlesnake population densities and/or lower prey densities (Beaupre, 1995; Madsen & Shine, 1993), or population differences with inland snakes genetically predisposed to grow larger and/or surviving longer (Forsman, 1993). Regardless of the cause, the implications of body size and temperature variation among adult male rattlesnakes at each of these sites result in minor differences in energetic needs to fuel maintenance metabolism, with snakes from each site needing less than one adult ground squirrel per year. Importantly, our metabolic calculations are only estimates of maintenance metabolism and do not encompass energetically costly activities including digestion and movement through the environment for mate‐seeking, predator avoidance, and ambush site selection (Beaupre, 1996, 2008). Furthermore, energetic needs of female rattlesnakes to produce a litter of offspring would be much higher (Beaupre, 2002; Beaupre & Duvall, 1998b). Population and sex differences in overall energy requirements could only be ascertained by collecting field metabolic data (e.g., Beaupre, 2008).

Climate change is generally predicted to have a negative effect on most ectotherm species, especially those at lower latitudes (Sinervo et al., 2010). However, it appears that small increases in ambient temperature may prove beneficial to rattlesnakes in central California because the overall thermal quality of all microhabitats is projected to increase at all field sites. With a larger proportion of daily *T*_mod_ falling within the *T*_set_ range, snakes will be less thermally constrained, choose among a wider range of ambush sites, and be active for a longer time during the day. Specifically, snakes will be able to emerge from overwintering earlier in the year and, in turn, wait until later months before going back into hiding. This may translate into additional opportunities to find resources such as mates and food, as well as longer annual active seasons, and could feasibly result in higher reproductive output in females and therefore increased population densities of rattlesnakes. It is ​also possible that summer temperatures may exceed *T*_set_ for longer periods of time during daylight hours (particularly at the inland sites), resulting in altered behavior, such as a shift to more crepuscular/nocturnal foraging. This could lead to utilization of alternate prey sources which in turn could start a cascade of ecological effects at the community level. If the metabolic rates of these snakes rise with increasing temperatures, they would need to obtain additional energetic resources. However, as our calculations and other studies have shown, the metabolic needs of these snakes are incredibly low (Beaupre, 1995; Beaupre & Duvall, 1998b; Beck, 1995), with current baseline maintenance energetic demands being met with less than a single large meal per year. Even if mean active *T*_b_ increased 1°C or 2°C along with ambient temperature, the annual caloric requirements for maintenance would still be met with a single large prey item. That said, evidence from this study suggests that *C. oreganus* is an accurate enough thermoregulator that overall small changes in ambient temperature will likely not dramatically shift the snakes' *T*_b_. These theoretical calculations are limited to the scope of energetic needs of the snakes from which they were calculated (i.e., resting, fasted snakes unable to thermoregulate in a metabolic chamber), so these results must also be considered in an ecological context. To fully understand the implications of climate change for rattlesnakes, we would need to take into account possible impacts on prey populations as well as changes in snake behavior as a response to changing temperatures, which may increase energetic needs beyond the scope of our models.

Overall, rattlesnakes are ideal model organisms for examining the physiological effects of climate on large‐bodied ectotherms. Their life‐history traits, simple behaviors, and metabolism are well studied, providing a strong foundation for examining their thermal ecology and implications of climate change on their energetic requirements. Large‐scale comparative studies among multiple populations of a given species can greatly enhance our understanding of the effects of anthropogenic climate change on biodiversity. While small increases in ambient temperature may prove thermally beneficial to rattlesnakes on the Central Coast of California, alterations in climates may affect rattlesnake environments in negative ways and have cascading effects within their biotic communities. It is apparent that in rattlesnakes, a wide‐ranging *T*_set_, plasticity in thermoregulatory behavior, and low energetic demands may help mitigate the changes in environmental temperatures these animals will experience, even across extremely variable habitat types.

## CONFLICT OF INTEREST

None declared.

## AUTHOR CONTRIBUTIONS

**Hayley L. Crowell:** Conceptualization (lead); data curation (lead); formal analysis (lead); investigation (equal); methodology (lead); project administration (lead); supervision (lead); visualization (lead); writing–original draft (lead). **Katherine C. King:** Data curation (equal); formal analysis (supporting); investigation (supporting); writing–original draft (supporting). **James M. Whelan:** Conceptualization (supporting); data curation (equal); formal analysis (supporting); investigation (supporting); methodology (supporting); writing–original draft (supporting). **Mallory V. Harmel:** Data curation (supporting); formal analysis (supporting); investigation (supporting); writing–original draft (supporting). **Gennesee Garcia:** Data curation (supporting); investigation (supporting); writing–original draft (supporting). **Sebastian G. Gonzales:** Data curation (supporting); investigation (supporting); writing–original draft (supporting). **Paul H. Maier:** Data curation (supporting); investigation (supporting); writing–original draft (supporting). **Heather M. Neldner:** Data curation (supporting); investigation (supporting); writing–review and editing (supporting). **Thomas Nhu:** Data curation (supporting); investigation (supporting); writing‐original draft (supporting). **John T. Nolan:** Data curation (supporting); investigation (supporting); writing–original draft (supporting). **Emily N. Taylor:** Conceptualization (lead); data curation (supporting); funding acquisition (lead); methodology (equal); resources (lead); supervision (supporting); validation (lead); writing–review and editing (lead).

## Supporting information

Supplementary Material

## Data Availability

All data associated with this project can be found on Dryad (https://doi.org/10.5061/dryad.mpg4f4qzv).
