# Supplementary material for: Thermal ecology and baseline energetic requirements of a large‐bodied ectotherm suggest resilience to climate change
Source: Ecol Evol. 2021 May 7;11(12):8170–82. doi: 10.1002/ece3.7649 (PMC8216919; doi:10.1002/ece3.7649)
Supplement: Supplementary file 1 — Supplementary Material [file ECE3-11-8170-s001.docx]

#### Supplementary Tables:

**Table S1.** Abbreviations and definitions of frequently used thermal ecology and metabolism-related variables.

| ***Abbreviation*** | ***Definition*** |
| --- | --- |
| T_b_ | body temperature (°C) |
| T_mod_ | temperature (°C) of rattlesnake physical model |
| T_set_ | preferred body temperature, usually represented as a 50% interquartile range from a group of body temperatures selected by an organism or set of organisms in a laboratory thermal gradient (°C) |
| d_b_ | thermoregulatory accuracy, the absolute value of the difference between field measured T_b_ and the animal’s T_set_ |
| d_e_ | habitat thermal quality, the absolute value of the difference between field-measured T_mod_ and the animal’s T_set_ |
| SMR | standard metabolic rate, the metabolic rate of a resting, fasted ectotherm at a specific body temperature |
|  |  |

**Table** **S2**. Environmental descriptions of four study sites on the Central Coast of California.

| ***Site*** | ***Average Annual Temp. (^o^C)*** | ***Average Annual Rainfall (cm)*** | ***Elevation (m)*** | ***Lat/Long*** | ***Dominant Vegetation*** | ***Human Use*** | ***Sources*** |
| --- | --- | --- | --- | --- | --- | --- | --- |
| **CR**  **(Inland)** | -9.4 - 46.7 | 18 - 23 | 671 | 35.1658°N  119.8696°W | Chaparral, Oak  Savanna, and Grassland Plains | Cattle grazing | Chimineas Ranch Foundation, 2019; TWC Product and Technology, 2019 |
| **SG**  **(Inland)** | -5 - 37.7 | 38 | 290 - 793 | 34.6928°N  120.0406°W | Chaparral, Oak  Savanna, and Grassland Plains | Private reserve partially used for cattle grazing | University of California Reserve System: Sedgwick Reserve, 2019 |
| **MDO**  **(Coastal)** | 5 - 20.6 | 0 - 9.4 | 0 - 411 | 35.2639°N 120.8632°W | Coastal Scrub, Chaparral | Frequent trail use by hikers | Capehart et al., 2016; Weatherforyou.com, 2019 |
| **VAFB**  **(Coastal)** | 5.5 - 21.7 | 0 - 8.1 | 156-700 | 34.7420°N  120.5724°W | Coastal Scrub | Cattle farming and occasional military traffic | Underwood et al., 2003; Weatherspark.com, 2018 |

TWC Product and Technology LLC. (2019) The Weather Channel: Carrizo Plain National Monument Monthly Weather, IBM Cloud. <https://weather.com/weather/monthly/l/CABLCARII:13:US>

Weatherforyou. (2019). Daily normals for Montana De Oro State Park, CA. Weatherforyou. https://www.weatherforyou.com/reports/index.php?forecast=norms&zipcode=&pands=&place=montana+de+oro+state+park&state=ca&country=us&day=all%2Cmonth&month=12

Weather Spark. (2018) Average weather at Lompoc, Vandenberg Air Force Base, California, USA. Cedar Lake Ventures, Inc. https://weatherspark.com/y/145288/Average-Weather-at-Lompoc-Vandenberg-Air-Force-Base;-California;-United-States-Year-Round

**Table S3.** Summary of which Pacific rattlesnake (*Crotalus oreganus*) populations were studied during which years for field active body temperature (T_b_) and metabolic rate experiments.

| *Site* | *Years Studied* | *No. of Individuals* |
| --- | --- | --- |
| CR (inland) | 2010  2017 | 22  1 |
| SG (inland) | 2015  2017 | 27  1 |
| MDO (coastal) | 2014  2017 | 11  4 |
| VAFB (coastal) | 2012  2013  2017 | 13  5  1 |

**Table S4:** Summary results of the repeated-measures ANOVA for the effects of site, month, and time of day on overall mean d_e_ of four field sites occupied by four distinct populations of Pacific rattlesnake (*Crotalus oreganus*) on the Central Coast of California.

| ***Predictor*** | ***F*** | ***df*** | ***P*** |
| --- | --- | --- | --- |
| Site | 31.29 | 3 | <0.0001* |
| Month | 121.67 | 11 | <0.0001* |
| Time | 64.14 | 23 | <0.0001* |
| Month*Time | 2.56 | 253 | <0.0001* |
| Month*Site | 4.98 | 33 | <0.0001* |
| Time*Site | 0.83 | 69 | 0.84 |
| Month*Time*Site | 0.33 | 759 | 1.00 |

**Table S5.** Summary of masses and field active body temperatures (T_b_) of Pacific rattlesnakes (*Crotalus oreganus*) used for standard metabolic rate estimates for each field site. Values are means ± 1 SEM.

| ***Site*** | ***n*** | ***Mean mass (g)*** | ***Mean T_b_ (℃)*** | ***T_b_ Range (℃)*** |
| --- | --- | --- | --- | --- |
| **CR (inland)** | 23 | 795.48 ± 53.91 | 23.39 ± 0.23 | 3.50 – 37.16 |
| **SG (inland)** | 28 | 720.30 ± 49.25 | 23.46 ± 0.20 | 5.14 – 69.91 |
| **MDO (coastal)** | 15 | 475.24 ± 42.05 | 20.93 ± 0.27 | 7.00 – 37.17 |
| **VAFB (coastal)** | 19 | 480.51 ± 21.77 | 21.80 ± 0.25 | 8.00 – 45.50 |

**Table S6.** Proportion of hourly physical model temperatures from June 2017 to June 2018 across four sites on the Central California Coast that fall within the interquartile T_set_ range of the Pacific rattlesnake (*Crotalus oreganus*) currently and with a 1 °C and 2 °C increase in ambient temperature. Also note the year in which these temperature increases are expected to occur varies by site. Years were extrapolated using the most conservative RCP model (4.5).

| ***Site*** | ***Microhabitat*** | ***Current (2017) Prop. of Temps within T_set_*** | ***1***°C ***increase***  ***(year expected)*** | ***2***°C ***increase***  ***(year expected)*** |
| --- | --- | --- | --- | --- |
| CR  (Inland) | Burrow  Shaded  Exposed | 0.07  0.05  0.07 | 0.11 (2030)  0.06 (2030)  0.09 (2030) | 0.14 (2052)  0.06 (2052)  0.09 (2052) |
| SG  (Inland) | Burrow  Shaded  Exposed | 0.03  0.08  - | 0.05 (2030)  0.08 (2030)  - | 0.07 (2050)  0.09 (2050)  - |
| MDO  (Coastal) | Burrow  Shaded  Exposed | 0.00  0.03  0.10 | 0.00 (2047)  0.04 (2047)  0.10 (2047) | 0.00 (2055)  0.05 (2055)  0.10 (2055) |
| VAFB  (Coastal) | Burrow  Shaded  Exposed | 0.02  0.07  0.08 | 0.04 (2030)  0.07 (2030)  0.08 (2030) | 0.06 (2053)  0.08 (2053)  0.08 (2053) |

**Table S7.** Preferred body temperatures (T_set_) from *Crotalus* as well as other viper species. Notes indicate any discrepancies or points the authors thought were relevant to their study.

| ***Species*** | ***Study*** | ***T_set_ (***°C***)*** | ***n*** | ***Notes*** |
| --- | --- | --- | --- | --- |
| ***Bothrops insularis*** | Bovo et al., 2012 | 20.4-26.3 | 85 | Interquartile range in winter and summer |
| ***Crotalus atrox*** | Brattstrom, 1965 | 27.4 | 8 | - |
| ***Crotalus cerastes*** | Moore, 1978 | 25.8 | 37 | *C. mitchellii* and *C. cerastes* were active at different times in the study to minimize competition. April-December. |
| ***Crotalus horridus*** | Brown, 1982 | 26.9 | 5 | - |
| ***Crotalus mitchellii*** | Moore, 1978 | 31.2 | 31 | April-December |
| ***Crotalus oreganus*** | *This study* | 26.3-32.3 | 51 | - |
| ***Crotalus viridis oreganus*** | Brattstrom, 1965  Moore, 1969 | 28.9  28-31 | 11  6 | - |
| ***Crotalus spp.*** | Brattstrom, 1965 | 29.7 | *atrox=8*  *cerastes=15*  *enyo=1*  *horridus=5*  *mitchellii=3*  *pricei=5*  *ruber=1*  *scutulatus=4*  *viridis=11*  *willardi=1* | 56 individuals recorded across 11 different species |
| ***Sistrurus catenatus*** | Harvey & Weatherhead, 2010 | 30-33.6 | 34 | - |
| ***Vipera aspis*** | Guillon et al., 2014 | 33 | 16 | - |

Brown, W.S., Pyle, D.W., Greene, K.R., & Friedlaender, J.B. (1982). Movements and temperature relationships of Timber Rattlesnakes (*Crotalus horridus*) in Northeastern New York. Journal of Herpetology. 16: 151–161. https://doi.org/10.2307/1563808

Guillon, M., Guiller, G., DeNardo, D.F., & Lourdais, O. (2014). Microclimate preferences correlate with contrasted evaporative water loss in parapatric vipers at their contact zone. Canadian Journal of Zoology. 92: 81-86. https://doi.org/10.1139/cjz-2013-0189

Harvey, D. S. & Weatherhead, P. J. (2010). Habitat selection as the mechanism for thermoregulation in a northern population of massasauga rattlesnakes (*Sistrurus catenatus*). Ecoscience 17: 411–419. https://doi.org/10.2980/17-4-3363

#### Supplementary Figures:

**Figure S1.** Median preferred body temperatures (T_set_) of Pacific rattlesnakes (*Crotalus oreganus*) from each site with 25^th^ % and 75^th^ % quartiles. There are no significant differences in T_set_ among the four sites after taking into account size, sex, presence of internal radio transmitter, and season. The mean T_set_ of all populations was 29.22 ± 0.92°C with an interquartile range of 26.28 ± 1.01°C - 32.34 ± 0.84°C.

A

Mean Daily Metabolic Rate (ml O_2_ day^-1^)


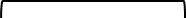


Site

B

**Figure S2.** Mean daily standard metabolic rate estimates (ml O_2_ day^-1^) of adult, male Pacific rattlesnake (*Crotalus oreganus*; N = 85; CR = 23, SG = 28, MDO = 15, VAFB = 19) from four different populations (two inland, two coastal) in Central California (calculations from Beaupre & Duvall, 1998). Snakes from inland sites have significantly higher metabolic rates than snakes from coastal sites. Different letters represent significant differences between means and standard error bars represent ±1 SEM.

**Figure S3**. Mean monthly Pacific rattlesnake (*Crotalus oreganus*) physical model temperatures (T_mod_) for each of the three microhabitat types (Burrow, Shaded, Exposed) at current climatic conditions (black line), with a 1˚C (dark grey line) and 2˚C (light grey line) increase in ambient temperature for four sites (inland: CR, SG; coastal: MDO, VAFB) on the Central Coast of California. Grey bars represent the preferred body temperature (T_set_) of *Crotalus oreganus*.

#### Supplemental Methodology:

*Thermal Gradient Construction & Data Collection*

The gradient consisted of three lanes (250 x 20 x 25cm each), separated by solid, wooden dividers, and lined with approximately 3 cm of sand to avoid the possibility of snakes getting burned on the metal floor of the trial arena. We designed the gradient to have closed water circuits running at both ends, with a water heater (Stiebel Eltron model no. SHC4, Holzminden, Germany*)* on the hot side and a water cooler on the cold side (ActiveAQUA Refrigerateur model no. AACH10, Petaluma, CA, USA). The water passed through copper pipes attached to the underside of the metal floor of the gradient; the pipes were covered with thick fiberglass insulation and dense foam boards on the bottom to reduce heat loss to the room. The distance between pipes increased as they approached the center point of the gradient to ensure that the heat transfer became more dispersed in the center.

We inserted thermocouples (model no. 5SRTC-TT-K-40-72, Omega Engineering, Egham, Surrey, United Kingdom) into the snakes’ cloacae and held them in place via surgical tape wrapped around the base of the tail. Thermocouples were connected to a data logger (model no. RDXL4SD, Omega Engineering, Egham, Surrey, United Kingdom) that automatically recorded the snakes’ internal body temperatures every ten minutes. The gradient was located in a room with consistent lighting and temperature (between 22 - 25°C) to reduce the effects of diel activity patterns and circadian rhythm on behavior, as trials were constantly running in order to maximize the number of snakes tested. Once snakes finished their trial, they were removed, and substrate was mixed and cleaned as needed to reduce the effects of scent on the behavior of new trial animals.

*Animal collection:*

We captured rattlesnakes at their respective field sites and transferred them to the laboratory where morphological data (i.e., SVL, mass) were recorded. They were housed individually in 71.12 x 60.96 x 30.48cm enclosures (Model V221, LLL Reptile and Supply Company, Inc., Vista, California, USA). Within 1-2 days of capture, rattlesnakes were then surgically implanted with Holohil radio-transmitters (models SB-2, 5.2g and SI-2,11g, 13.5 g; assigned to snakes based on their mass; Holohil Systems Ltd., Carp, Ontario, CA) following the procedures of Claunch et al. (2017). Additionally, snakes were implanted with Thermochron iButtons (DS1922L-F5 and DS1921G-F5 models, Maxim Integrated Products Inc., Rio Robles, San Jose, CA), which were set to record field active body temperatures (T_b_) every hour. Immediately following surgery, acrylic paint was injected into the three proximal rattle segments in unique color combinations to allow for accurate field identification of individual snakes (Taylor & DeNardo, 2005; Lind et al., 2010; Putman et al., 2013). Snakes were then returned to their enclosures and allowed to recover in the lab for at least 24 hours prior to release.

*Physical Model Construction & Validation*

Physical models were 40cm x 2.5cm copper pipes filled with water and capped at one end with a screw-on PVC cap and at the other with a welded-on copper cap (Bakken, 1992; Lutterschmidt & Reinert, 2012). Each pipe was painted with a base layer of mixed Rust-Oleum © “Matte Burlap” and “Multicolor textured” tan spray paints. A dorsal diamond pattern similar to that of a live *C. oreganus* was painted in “Matte bittersweet” dark brown (Rust-Oleum Corporation, Vernon Hills, IL, USA). A Thermochron iButton temperature data logger (model no. DL1922) was suspended in the center of each pipe by a 3D-printed, plastic mount that prevented the iButton from touching any of the surrounding pipe walls to avoid direct conduction from the metal pipe to the data logger (Bakken, 1992).

We validated snake physical models using two average-sized (570g, 740g) adult rattlesnake carcasses of similar width that had recently been found dead on the road. Snake carcasses and three physical models (implanted with iButtons in the same fashion as their respective experimental field counterparts, programmed to take a temperature reading every 10 minutes) were cooled in a fridge for 1 hour and then placed outside on leaf litter, under direct sunlight for 2.5 h. Physical models tended to heat slightly faster than snake carcasses but model temperature and snake carcass temperatures were highly correlated and validated using a Pearson linear regression (snake carcass = 0.63 + (1.05 * Physical model ), r^2^ = 0.97, F_1,14_ = 496.21, p<0.0001).

*Energetics: SMR Calculations*

The T_b_ for each individual was recorded hourly or bi-hourly with an iButton implanted within the lower third body cavity of the snake (Taylor & DeNardo, 2005; Lind et al., 2010; Putman et al., 2013). For snakes where T_b_ was recorded bihourly, missing hourly temperatures were interpolated by taking the average of the next closest body temperatures before and after the missing hourly value. T_b_ data were then sorted into five different time-of-day blocks based on the metabolic calculation methods established by Beaupre and Duvall (1998b), which take into account the hourly variation in rattlesnake metabolic rates base due to circadian rhythms. The hours of 1100-1600 were not included in the original literature (Beaupre & Duvall, 1998b), so for our study we assigned temperatures from the hours of the 1100-1400 time block to the 0800-1100 time block and 1400-1600 h to the 1600-2000 time block. In the established equation, “X” values are fitted constants established for each metabolic time block. Snake mass, collected during the initial radio-tagging procedure, and field active T_b_ were inputted into the appropriate equation during the aligning time block and the inverse log of the resulting value taken as the volume of oxygen (ml of O_2_ hour^-1^) that was consumed by the individual.

Lind, C., Husak, J. F., Eikenaar, C., Moore, I. T., & Taylor, E. N. (2010). The relationship between plasma steroid hormone concentrations and the reproductive cycle in the Northern Pacific rattlesnake, *Crotalus oreganus.* General and Comparative Endocrinology. 166: 590-599. https://doi.org/10.1016/j.ygcen.2010.01.026

Putman, B. J., Lind, C., & Taylor, E. N. (2013). Does size matter? Factors influencing the spatial ecology of Northern Pacific rattlesnakes (*Crotalus oreganus oreganus*) in Central California. Copeia. 3: 485-492. https://doi.org/10.1643/CE-12-048

Taylor, E.N. & DeNardo, D.F. (2005). Reproductive ecology of Western Diamond-backed rattlesnakes (*Crotalus atrox*) in the Sonoran Desert. Copeia. 1: 152-158. https://doi.org/10.1643/CH-04-107R1
